# The Treatment of a Novel Epidermal Growth Factor Receptor (EGFR)-GRB2 Genetic Mutation Using Osimertinib in Metastatic Non-Small Cell Lung Cancer

**DOI:** 10.7759/cureus.38059

**Published:** 2023-04-24

**Authors:** Siddharth Ramanathan, Nathan Shen, Larry Kestin, Savitha Balaraman

**Affiliations:** 1 College of Medicine, Oakland University William Beaumont School of Medicine, Rochester Hills, USA; 2 Hematology and Oncology, Central Michigan University College of Medicine, Mount Pleasant, USA; 3 Radiation Oncology, Genesis Care, Troy, USA; 4 Hematology and Oncology, Michigan Healthcare Professionals, Royal Oak, USA

**Keywords:** tyrosine kinase inhibitors (tki), metastatic lung cancer, osimertinib, lung adenocarcinoma, egfr

## Abstract

Mutations in the epidermal growth factor receptor (EGFR) have been implicated in nearly one-third of non-small-cell lung cancers. For patients harboring non-traditional mutations, genomic and transcriptomic sequencing can help direct treatment. As cancer genomics evolves, novel driver mutations continue to be uncovered. We report on a unique EGFR-GRB2 fusion in a 48-year-old female never-smoker. This patient presented with stage IV lung adenocarcinoma (T2aN3M1) with metastatic disease in the iliac wing and liver. Despite systemic treatment, this patient continued to progress. On whole transcriptome sequencing, this patient was found to have a novel EGFR-GRB2 RNA fusion transcript similar to other EGFR fusions described in the literature. After treatment with osimertinib, this patient experienced remarkable clinical and radiological improvements. We believe that, especially for patients with metastatic lung cancer, the presence of novel driver mutations should be investigated. Potentially, patients harboring similar mutations may demonstrate analogous improvements with targeted treatment using the most recent generation of tyrosine kinase inhibitors.

## Introduction

The epidermal growth factor receptor (EGFR) has been well studied, with significant implications for carcinogenesis and anti-cancer therapeutics [1]. In lung cancers specifically, the most common mutations in the EGFR gene account for 32.3% of all non-small cell lung cancer (NSCLC), almost all of which occur on exons 19 or 21 of the EGFR transcript [2]. Anti-EGFR therapies targeting these pathogenic mutations can be broadly classified into two major groups: small-molecule tyrosine kinase inhibitors (TKIs) and monoclonal antibodies (mAbs) [3]. Several small-molecule EGFR inhibitors have gained Food and Drug Administration (FDA) approval for the treatment of lung cancer, including gefitinib, erlotinib, afatinib, osimertinib, dacomitinib, and mobocertinib. Anti-EGFR monoclonal antibodies such as amivantamab-vmjw have also been approved for the treatment of lung cancer patients who possess unique mutations that are not as susceptible to treatment with TKIs, such as exon 20 insertions [4].

Despite these breakthrough drugs, current recommendations confine the utilization of TKIs as first-line therapies to patients possessing a relatively narrow subset of mutations. These recommendations, in conjunction with the development of treatment resistance mutations, encumber the ability to reliably treat mutant EGFR lung adenocarcinomas in the long term. The expansion of the known set of actionable mutations is a distinct area of interest that has the potential to increase the treatment options for NSCLC by broadening the clinical utilization of EGFR TKIs [5]. 

This case study identifies one such mutation: a novel EGFR-GRB2 RNA fusion detected using whole transcriptome sequencing (WTS) that demonstrated a remarkable response to osimertinib, a third-generation EGFR TKI, in a patient with diffusely metastatic pulmonary adenocarcinoma with multiple cerebral metastases.

Background

A small selection of gene mutations is present in the vast majority of lung adenocarcinomas. These include mutations in the following major oncogenes: EGFR, ALK, KRAS, BRAF, NTRK 1/2/3, METex14, and RET. Patients with metastatic NSCLC harboring actionable mutations are treated with specific targeted therapies, including TKIs and mAbs. EGFR mutations are the most common oncogenic mutations implicated in lung adenocarcinoma, second only to KRAS mutations. This makes EGFR mutations an especially attractive target for anticancer therapeutics [5].

The EGFR is a member of the ErbB family of transmembrane receptor tyrosine kinases that are activated by the binding of specific ligands, such as epidermal growth factor (EGF) and transforming growth factor α (TGF-α), to the extracellular ligand-binding domain. Upon activation, the cytoplasmic portion of the EGFR undergoes a transition from an inactive monomeric form to an active homodimeric/heterodimeric conformation, thereby triggering autophosphorylation of tyrosine residues in the C-terminal domain of the cytoplasmic portion of the peptide chain. Receptor activation through autophosphorylation by intrinsic tyrosine kinase activity initiates several signal transduction pathways. The downstream RAS-RAF-MAPK, JAK/STAT, and PI3K/AKT pathways trigger a cascade of signaling events that are responsible for cellular proliferation, cellular differentiation, and upregulation of the EGFR [6].

Specific mutations in the EGFR gene have been linked to increased expression of various oncogenic proteins in lung adenocarcinomas and have thus become a major target of various TKIs. EGFR mutations have been identified as being highly specific to NSCLC; these mutations are found in 38% of NSCLC adenocarcinomas compared to only 12% of the non-small cell, non-adenocarcinomas. This association makes the EGFR receptor a particularly appealing target in the treatment of NSCLC [5-6]. 

Several studies have demonstrated that first-generation TKIs, such as gefitinib and erlotinib, are more effective than traditional chemotherapy (pemetrexed plus cisplatin) in the treatment of lung cancer [7]. More recently, second-generation EGFR-TKIs have been developed to treat NSCLC. While these medications have been proven to increase progression-free survival (PFS) in patients with NSCLC compared to treatment with either first-generation TKIs or traditional chemotherapy, second-generation TKIs result in an increased risk for side effects compared to first-generation molecules [8]. 

Patients with EGFR mutations in exons 18, 19, and 21 respond well to first- and second-generation TKIs. Unfortunately, acquired resistance while on continuous treatment with an EGFR TKI remains a significant challenge when treating patients with EGFR-mutant NSCLC. Typically, acquired resistance develops after a median of 9.2-14.7 months of TKI therapy [9]. More than 60% of patients develop resistance to first- or second-generation TKIs through an acquired second-site mutation in the EGFR ATP-binding pocket of exon 20 at amino acid position 790 (T790M) [10].

To combat this resistance, third-generation EGFR TKIs have been developed. Osimertinib was the first third-generation EGFR inhibitor approved by the FDA in 2015 for its efficacy against lung adenocarcinomas harboring the EGFR T790M mutation. Importantly, osimertinib benefits from a relatively modest side effect profile, especially when compared to second-generation TKIs, while possessing reasonable central nervous system (CNS) penetration [11].

The recent findings in the FLAURA trial showed that osimertinib improves median PFS and overall survival (OS) in patients with previously untreated EGFR mutation-positive (exon 19 deletion or L858R) advanced NSCLC compared to those treated with a first-generation TKI [12]. Subsequently, osimertinib was approved by the FDA for use as adjuvant therapy after tumor resection in adult NSCLC patients or as first-line treatment in adult NSCLC patients with a metastatic disease whose tumors have EGFR exon 19 deletions or exon 21 L858R mutations. This technically limits the potential application of osimertinib in the treatment of lung adenocarcinomas [11-12]. To investigate the therapeutic range of this drug, the efficacy of osimertinib in patients with novel gene mutations should be explored. This case study illustrates the remarkable clinical efficacy of osimertinib in a patient with stage IV lung adenocarcinoma with multiple visceral metastases. This patient had a novel EGFR-GRB2 RNA fusion that may have played a role in the responsiveness of her late-stage cancer to osimertinib therapy. Informed consent was obtained from the patient’s estate to publish the patient’s course of cancer treatment. 

## Case presentation

In September of 2014, a 48-year-old female never-smoker was diagnosed with metastatic clinical stage IV (T2a N3 M1) adenocarcinoma of the lung with impending superior vena cava syndrome. At initial diagnosis, computed tomography (CT) and positron emission tomography (PET) scans revealed a 4.1 cm right upper lobe lung mass with bilateral mediastinal, ipsilateral hilar, and biopsy-proven contralateral cervical lymph node involvement. There were two distant metastases at the time of diagnosis: one in the liver and one in the anterior right iliac wing. The tumor tested negative for known driver mutations in ALK, BRAF, EGFR, ROS1, RET, and MET genes and was found to possess a 10% PD-L1 tumor proportion score via immunohistochemistry (IHC) based on Caris Life Science’s© next-generation sequencing (NGS) panel, satisfying the criteria for PD-L1 positivity. 

**Figure 1:**
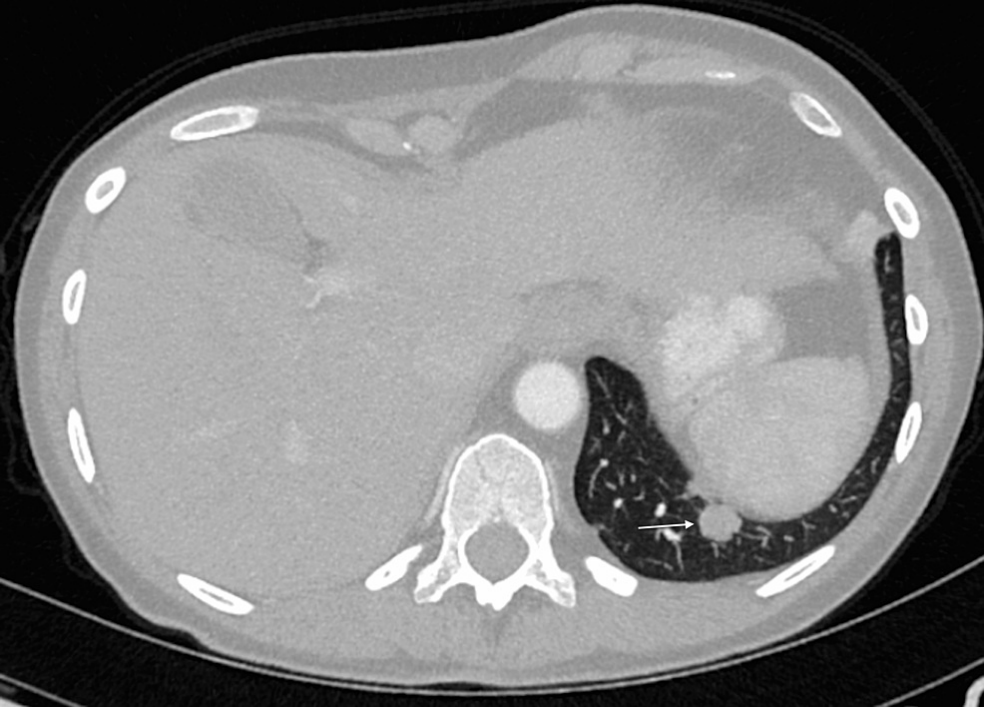
Axial CT demonstrating left lung base lesion in November 2019

**Figure 2:**
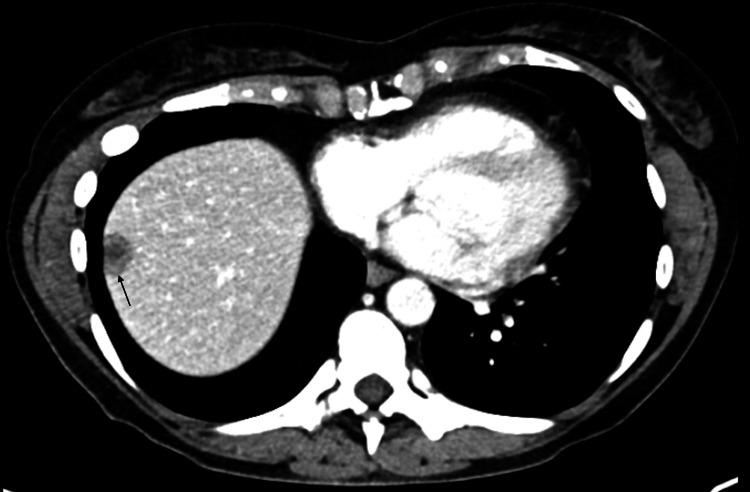
Axial CT demonstrating right hepatic lesion November 2019

The patient was initially treated with carboplatin, pemetrexed, and zoledronic acid with concomitant radiation therapy to the regions of thoracic involvement (60 Gy in 30 fractions), followed by maintenance pemetrexed. The patient progressed in February 2015 and was treated with sequential combination and single-agent therapies as well as immunotherapy with Nivolumab (based on her high tumor mutational burden [TMB] status). The patient also underwent palliative radiation to the anterior right anterior iliac wing in December 2017, receiving a total dose of 40 Gy in 20 fractions. In November 2019, almost five years after her initial diagnosis, the patient was found to have seven metastatic brain lesions, which were treated with Gamma Knife radiosurgery. In addition, there was evidence of dramatic systemic progression with multiple bilateral pulmonary nodules, progressive liver and bone metastases, and increasing celiac, retroperitoneal, and pelvic lymphadenopathy (Figures 1, 2). The patient developed progressively worsening shortness of breath and rib pain, which required increasing doses of analgesics.

**Figure 3:**
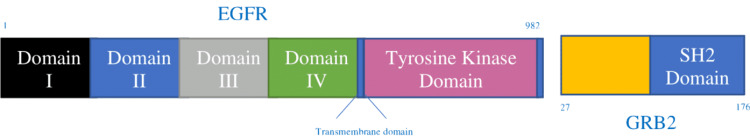
Diagrammatic representation of EGFR-GRB2 fusion

Given that the patient had failed multiple systemic therapies, a repeat biopsy of the liver was performed to identify the potential presence of novel driver mutations. Pertinent IHCs and NGS (a 592-gene DNA panel and whole transcriptome RNA sequence) were performed by Caris Life Sciences (Phoenix, AZ). The tumor was again negative for known oncogenic mutations in ALK, BRAF, EGFR, ROS1, MET, NTRK 1/2/3, ERBB2, KRAS, and RET genes. It was also microsatellite stable and PD-L1 negative by IHC (22c3) but with a high TMB. Interestingly, a novel EGFR-GRB2 fusion was detected in this tumor by whole transcriptome sequencing (Figure 3). While not previously reported in the literature, this fusion is in-frame and similar to other previously identified oncogenic EGFR mutations [13]. 

Despite her progressive metastatic cancer, the patient had a relatively preserved Karnovsky performance status and wanted to pursue aggressive treatment. She was therefore started on osimertinib based on the presence of a likely oncogenic EGFR fusion and the patient’s history of multiple metastatic brain lesions. 

The patient was made aware that osimertinib was approved by the FDA for first-line treatment of adult patients with EGFR exon 19 deletion (Ex19del) or Leu858Arg (L858R) EGFR mutations, alone or co-occurring with other EGFR mutations in locally advanced and metastatic NSCLC. She was also advised that her specific EGFR mutation was not studied in any trials and that her treatment was off clinical study and outside of FDA recommendations [11]. 

**Figure 4:**
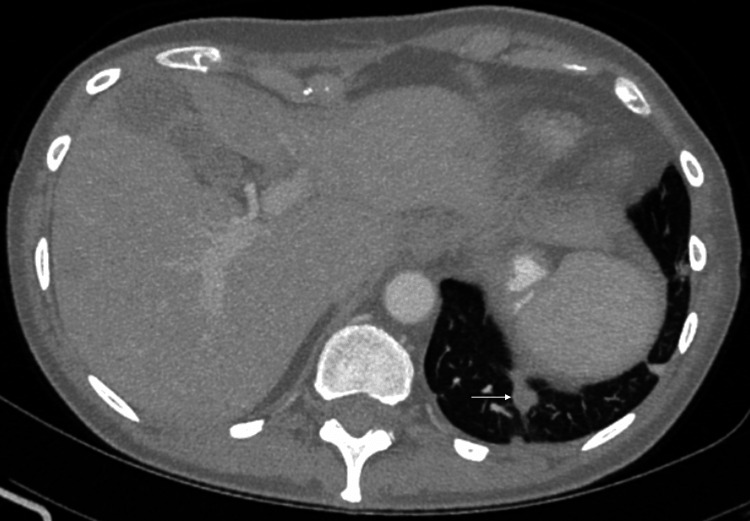
Repeat axial CT showing left lung base lesion demonstrating decreased diameter in February 2020

**Figure 5:**
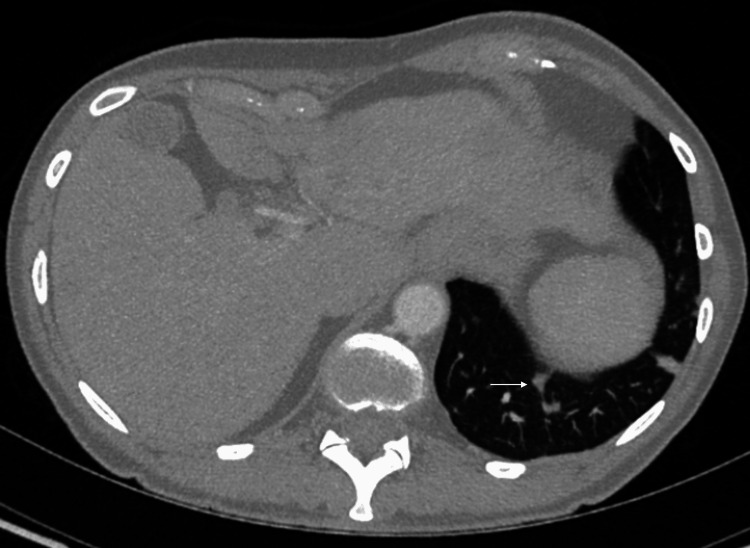
Repeat axial CT showing left lung base lesion demonstrating decreased diameter in May 2020

The patient was treated with osimertinib for nine months, between January 2020 and September 2020, with significant clinical and radiological responses. Her shortness of breath and rib pain improved dramatically, and her nausea resolved soon after. Radiologically, the patient’s pulmonary and hepatic nodules, bone metastases, and abdominal lymphadenopathy decreased, and the lymphangitic changes in the lungs resolved (Figures 4, 5). Subsequently, the patient was placed on maintenance pemetrexed, and in June of 2021, when her cancer progressed, the patient chose to enroll in hospice.

## Discussion

The EGFR-GRB2 fusion is significant due to its ability to activate downstream pathways without the binding of a ligand to the EGFR. Other rare EGFR fusions have been reported in lung adenocarcinomas, including cutaneous squamous cell carcinoma, colorectal carcinoma, and glioblastoma multiforme. In these studies of EGFR fusions, treatment with TKIs has shown clinical or preclinical improvement in tumor cell count and/or tumor burden [14].

Growth factor receptor-bound protein 2 (Grb2) is a cytoplasmic adaptor protein involved in intracellular signal transduction, cell cycle progression, and cell motility. The constitutively expressed GRB2 gene codes for the 25kDa Grb2 protein, which is composed of two SH3 domains and an Src homology 2 (SH2) domain. Grb2 binds to specific proline-rich and phosphotyrosine motifs on cytosolic receptors, such as EGFR, to interact with Son of Sevenless (Sos) proteins (guanine-nucleotide exchange factors), thereafter promoting RAS to induce a phosphorylation cascade that activates the mitogen-activated protein kinase (MAPK) cascade alongside various other downstream kinases such as ERK1 and ERK2. GRB2 mutations have been linked to an extensive list of cancers, such as breast cancer, thyroid cancer, leukemias, and hepatocellular carcinoma. Because of this oncogenic link, Grb2 inhibition has been a prime target to prevent the local invasion and metastasis of solid tumors [15]. Notably, in our patient’s novel fusion, exon 24 of EGFR is joined to exon 3 of GRB2, where the tyrosine kinase domain of EGFR and the SH2 domain of GRB2 is retained in the fusion. The SH2 domain binds specifically to tyrosine-phosphorylated sites of EGFR [16]. The GRB2 segment of the fusion transcript lacks an exon in the 3’ coding region, resulting in a shortened GRB2 isoform.

In the most recent phase III FLAURA trial, osimertinib demonstrated superior efficacy compared to its first-generation EGFR-TKI counterparts. Osimertinib hosts a more tolerable toxicity profile and possesses increased penetration through the blood-brain barrier compared to previous EGFR-TKIs [12]. The Bloom study found a greater cerebrospinal fluid (CSF) concentration of osimertinib and a decreased probability of CNS progression, thereby affirming the increased CNS penetrative potential of osimertinib compared to first-generation EGFR-TKIs [17]. This was the rationale for treatment with osimertinib for this patient with an atypical EGFR-GRB2 fusion. 

Several other third-generation irreversible EGFR-TKIs have been evaluated, including Avitinib, Olmutinib, and Nazartinib. Initial evaluations of Olmutinib have shown inferior safety and efficacy profiles compared to Osimertinib; two patients developed Steven-Johnson syndrome, and one patient developed fatal toxic epidermal necrolysis [18]. Although experiments with Avitinib have demonstrated adequate targeting of EGFR-active mutants with a relatively benign risk of side effects, these studies evaluate outcomes in animal models (although ongoing trials in China are evaluating the safety and clinical efficacy in NSCLC patients) [18]. A stage III trial comparing Nazartinib with first-generation EGFR-TKIs has since been withdrawn by the sponsor, although trials are still investigating combination therapies involving Nazartinib [19]. In sum, Osimertinib remains the only third-generation EGFR-TKI with FDA approval for the treatment of NSCLC, making it crucial to understand the efficacy of this medication in the treatment of NSCLCs harboring abnormal genomic profiles. 

## Conclusions

To our knowledge, this is the first-ever case report of a patient with an EGFR-GRB2 RNA fusion abnormality treated with osimertinib. The PFS of > 7 months with osimertinib in a patient with diffuse metastatic disease is encouraging in this poor prognostic subset of patients with advanced NSCLC. Based on this case report, we consider further exploration of the activity of osimertinib in unusual EGFR fusions to be warranted.
